# The Ubiquitin-specific Protease USP36 Associates with the Microprocessor Complex and Regulates miRNA Biogenesis by SUMOylating DGCR8

**DOI:** 10.1158/2767-9764.CRC-22-0344

**Published:** 2023-03-20

**Authors:** Yanping Li, Timothy S. Carey, Catherine H. Feng, Hong-Ming Zhu, Xiao-Xin Sun, Mu-Shui Dai

**Affiliations:** 1 Department of Molecular and Medical Genetics, School of Medicine, Oregon Health & Science University, Portland, Oregon.

## Abstract

miRNA biogenesis is a cellular process that produces mature miRNAs from their primary transcripts, pri-miRNAs, via two RNAse III enzyme complexes: the Drosha-DGCR8 microprocessor complex in the nucleus and the Dicer-TRBP complex in the cytoplasm. Emerging evidence suggests that miRNA biogenesis is tightly regulated by posttranscriptional and posttranslational modifications and aberrant miRNA biogenesis is associated with various human diseases including cancer. DGCR8 has been shown to be modified by SUMOylation. Yet, the SUMO ligase mediating DGCR8 SUMOylation is currently unknown. Here, we report that USP36, a nucleolar ubiquitin-specific protease essential for ribosome biogenesis, is a novel regulator of DGCR8. USP36 interacts with the microprocessor complex and promotes DGCR8 SUMOylation, specifically modified by SUMO2. USP36-mediated SUMOylation does not affect the levels of DGCR8 and the formation of the Drosha-DGCR8 complex, but promotes the binding of DGCR8 to pri-miRNAs. Consistently, abolishing DGCR8 SUMOylation significantly attenuates its binding to pri-miRNAs and knockdown of USP36 attenuates pri-miRNA processing, resulting in marked reduction of tested mature miRNAs. Induced expression of a SUMOylation-defective mutant of DGCR8 inhibits cell proliferation. Together, these results suggest that USP36 plays an important role in regulating miRNA biogenesis by SUMOylating DGCR8.

**Significance:**

This study identifies that USP36 mediates DGCR8 SUMOylation by SUMO2 and is critical for miRNA biogenesis. As USP36 is frequently overexpressed in various human cancers, our study suggests that deregulated USP36-miRNA biogenesis pathway may contribute to tumorigenesis.

## Introduction

miRNAs are a major class of endogenous small noncoding RNAs of approximately 22 nucleotides in length that are potent posttranscriptional regulators of gene expression (1, 2). They are generally transcribed by RNA polymerase II as capped and polyadenylated transcripts called primary miRNAs (pri-miRNA), which are processed to precursor miRNAs (pre-miRNA) by the microprocessor complex consisting of the RNAse III enzyme Drosha and the double-stranded RNA-binding protein (RBP) DGCR8 (3–5). Pre-miRNAs are then exported to the cytoplasm by exportin 5 where they are finally processed by the RNAse III enzyme Dicer to mature miRNAs (3–5). miRNAs complementarily bind to target mRNAs, resulting in the degradation of target mRNAs and/or the repression of their translation (1, 2). Consequently, miRNAs have emerged as key regulators of gene expression that shape many cellular processes, regulate development pathways, and determine cell identity and cell fate (1, 6, 7). Not surprisingly, aberrant miRNA biogenesis is associated with various human diseases including cancers (8, 9). Therefore, the miRNA biogenesis pathway is tightly controlled during normal cell homeostasis and their deregulation plays a significant role in tumor formation and progression.

Recent structural studies have shown that the microprocessor complex contains two DGCR8 molecules and one Drosha molecule, forming a trimeric complex that determines the cleave site in pri-miRNAs (5, 10, 11). The two DGCR8 molecules bind to the two dsRNA binding domains (dsRBD) of Drosha, respectively, via their C-terminal tail (CTT) region (5, 10, 11). In addition, miRNA biogenesis is regulated by a number of RBPs (3, 4, 7, 12). For example, LIN28A inhibits pre-let-7 processing by binding to the pre-miRNA terminal loop (TL) whereas LIN28B binds to pri-let-7 transcripts and inhibits their processing by microprocessor (13, 14). hnRNP A1 and KSRP bind to pri-miRNA TLs and facilitate microprocessor-mediated pri-miRNA processing (15, 16). Tumor suppressor protein p53 and SMAD have been shown to regulate miRNA biogenesis by associating with auxiliary factors such as the DEAD box RNA helicase p68, thereby modulating miRNA expression profile in cancers (17, 18). The RBP DDX1 promotes the maturation of a subset of pri-miRNA and inhibits ovarian cancer progression by interacting with the Drosha microprocessor (19). YAP regulates miRNA biogenesis in a cell density–dependent manner by sequestering the DEAD box RNA helicases p72 and may mediate wild-spread miRNA repression observed in cancer (20). Also, posttranslational regulation of proteins in the miRNA biogenesis pathways has begun to be revealed. For example, phosphorylation of DGCR8 promotes its stability and microprocessor activity and increases miRNA levels (21). mTOR stimulates the expression of MDM2 oncoprotein, which ubiquitinates Drosha and leads to the inhibition of miRNA biogenesis in response to energy deprivation (22). DGCR8 has also been shown to be modified by SUMOylation which modulates its binding to pri-miRNAs (23). However, how DGCR8 is SUMOylated in cells is still unknown.

Here, we report that USP36, a nucleolar ubiquitin-specific protease essential for ribosome biogenesis and cell growth and frequently overexpressed in cancer (24–28), acts as a novel posttranslational regulator of DGCR8. USP36 interacts with the DGCR8-Drosha complex and promotes DGCR8 SUMOylation specifically by SUMO2. We show that USP36-mediated SUMOylation of DGCR8 is critical for DGCR8 binding to target pri-miRNAs and miRNA biogenesis. Consistently, depletion of USP36 markedly reduced the levels of tested mature miRNAs and induced overexpression of a SUMOylation-defective mutant of DGCR8 suppressed cell proliferation. Our results suggest a novel role for USP36 in gene regulation by modulating miRNA biogenesis.

## Materials and Methods

### Cell Culture, Plasmids, Antibodies, and Reagents

Human lung small cell carcinoma H1299 cells, human cervical carcinoma HeLa cells, human osteosarcoma U2OS cells, and human embryonic kidney epithelial 293 cells were cultured in DMEM supplemented with 10% (vol/vol) FBS, 50 U/mL penicillin, and 0.1 mg/mL streptomycin at 37°C in a 5% CO_2_ humidified atmosphere as described previously (27–29). Human lung fibroblast IMR-90 cells were cultured in DMEM supplemented with 15% (vol/vol) FBS and MEM non-essential amino acids. These cell lines were obtained from ATCC. Cell lines were passaged less than 30 times for maximal 2 months and routinely monitored for *Mycoplasma* contamination. Manufacturers performed authentication through short tandem repeat profiling.

Flag-tagged full-length USP36 [wild-type (WT) and the C131A mutant], its deletion mutants, and V5-tagged USP36 were described previously (27, 28). V5-Drosha-pCK, Flag-Drosha-pCK, pCK-Drosha-Flag mutants, V5-DGCR8-pCK, and Flag-DGCR8-pCK plasmids were kindly provided by Dr. Narry Kim (Seoul National University, Seoul, Republic of South Korea). All Flag-tagged DGCR8 deletion mutants were constructed by inserting PCR products into pcDNA3-2Flag vector. Flag-DGCR8^K707R^-pCK, and Flag-DGCR8^K259R^-pCK, Flag-DGCR8^K426R^-pCK, Flag-DGCR8^K259R/K707R^-pCK, Flag-DGCR8^K426R/K707R^-pCK and Flag-DGCR8^K259R/426R/K707R^-pCK (Flag-DGCR8^3KR^) mutants were generated by site-directed mutagenesis using QuikChange Kit (Agilent). Flag-DGCR8 and Flag-DGCR8^3KR^ cDNAs were also cloned into pcDNA4-TO vector (Life Technologies) to generate pcDNA4-TO-Flag-DGCR8^WT^ and pcDNA4-TO-Flag-DGCR8^3KR^ vectors. His-tagged SUMO1, SUMO2, ubiquitin (Ub) plasmids were described previously (27, 30).

Anti-Flag (M2, F3165, Sigma, RRID: AB_259529), anti-V5 (R960-25, Life Technologies, RRID:AB_2556564), anti-Drosha (A301-886A, Bethyl Laboratory, RRID:AB_1309798), anti-DGCR8 (A302-468A, Bethyl Laboratory, RRID:AB_1944223), anti-Drosha (sc-393591, Santa Cruz Biotechno-logy, RRID:AB_2732793), anti-Ub (sc-9133, Santa Cruz Biotechnology, RRID:AB_2180553), anti-Nop58 (A302-719A, Bethyl Laboratory, RRID:AB_10755121), anti-nucleostemin (NS; sc-166460, Santa Cruz Biotechnology, RRID:AB_2110096), anti-USP36 (14783-1-AP, Proteintech, RRID:AB_2213357) antibodies, anti-tubulin (66240-1-Ig, Proteintech, RRID:AB_2881629) were purchased. Rabbit anti-USP36 serum was provided by Dr. Masayuki Komada (Tokyo Institute of Technology, Tokyo, Japan; refs. 24, 28).

### Transfection, Immunoblot, and Co-immunoprecipitation Analyses

Cells were transfected with plasmids using Lipofectamine 2000 (Life Technologies) or TransIT-LT1 reagents (Mirus Bio Corporation) following the manufacturers’ protocol. Cells were harvested at 36–48 hours posttransfection and lysed in NP40 lysis buffer consisting of 50 mmol/L Tris-HCl (pH 8.0), 0.5% Nonidet P-40, 1 mmol/L Ethylenediaminetetraacetic acid (EDTA), 150 mmol/L NaCl, 1 mmol/L phenylmethylsulfonyl fluoride (PMSF), 1 mmol/L Dithiothreitol (DTT), 1 μg/mL pepstatin A, and 1 mmol/L leupeptin. Equal amounts of total protein were used for immunoblot (IB) analysis. Co-immunoprecipitation (co-IP) was conducted as described previously (28). Bound proteins were detected by IB using antibodies as indicated in figure legends.

### Gene Knockdown by RNAi

Lentiviral vectors encoding short hairpin RNAs (shRNA) against USP36 were purchased (Open Biosystems). The shRNA sequences were 5′-GCGGTCAGTCAGGATGCTATT-3′ (USP36 shRNA-1), 5′-CGTCCGTATATGTCCCAGAAT-3′ (USP36 shRNA-2). The plasmids were transfected with VSVG, pLP1, pLP2 plasmids into 293FT cells using Calcium Chloride (Promega). The viruses were then used to infect cells in the presence of polybrene (6 μg/mL). The cells were harvested at 72 hours after transduction for IB analysis. For siRNA-mediated knockdown, the 21-nucleotide siRNA duplexes with a 3′ dTdT overhang were synthesized by Dharmacon Inc. The target sequences for DGCR8 was 5′-CAUCGGACAAGAGUGUGAU-3′ and the target sequences for Drosha was 5′-CGAGUAGGCUUCGUGACUU-3′. The control scramble RNA was described previously (30). DGCR8 siRNA pool (sc-60529) and Drosha siRNA pool (sc-44080) were also purchased from Santa Cruz Biotechnology. These siRNA duplexes (100 nmol/L) were introduced into cells using Lipofectamine 2000 (Invitrogen) following the manufacturer's protocol.

### *In Vivo* Ubiquitination and SUMOylation Assays

*In vivo* ubiquitination and SUMOylation assays under denaturing conditions were conducted using a Ni^2+^-NTA pulldown (PD) method as described previously (29–31). For ubiquitination assay, cells were transfected with His-Ub and indicated plasmids and treated with 40 μmol/L MG132 for 6 hours before harvesting. The cells were harvested at 48 hours after transfection and 20% of the cells were used for direct IB and the rest of cells were subjected to Ni^2+^-NTA PD under denaturing conditions. The bead-bound proteins were analyzed using IB. For SUMOylation assay, cells were transfected with His-SUMO1 or His-SUMO2 and indicated plasmids followed by Ni^2+^-NTA PD under denaturing conditions as above.

### qRT-PCR

Total RNA was isolated from cells using Qiagen miRNeasy mini Kit (Qiagen). Reverse transcriptions were performed using RevertAid RT Reverse transcription Kit (K1691, Thermo Fisher Scientific). Quantitative real-time PCR was performed on an ABI StepOne real-time PCR system (Applied Biosystems) using TaqMan miRNA assay kits (Applied Biosystems) for detecting mature miRNAs following the manufacturer's protocol and SYBR Green Mix (Thermo Fisher Scientific) for detecting pri-miRNAs. All reactions were carried out in triplicate. Relative gene expression was calculated using the ΔCτ method following the manufacturer's instruction. The primers used for detecting pri-miRNAs were as follows: 5′-CTCGCTTCGGCAGCACA-3′ and 5′-AACGCTTCACGAATTTGCGT-3′ for U6; 5′-TTTTGTTTTGCTTGGGAGGA-3′ and 5′-AGCAGACAGTCAGGCAGGAT-3′ for pri-miR-21; 5′-CATCTACTGCCCTAAGTGCTCCTT-3′ and 5′-GCTTGGCTTGAATTATTGGATGA-3′ for pri-miR-20a; 5′-TCTCAAGTGCATCCTGAAGAGTTC-3′ and 5′-AAACTACTACCTCAGCCTGGAATCA-3′ for pri-let-7g; 5′-CCTGGATGTTCTCTTCACTG-3′ and 5′-GCCTGGATGCAGACTTTTCT-3′ for pri-let-7a-1; 5′-AGCTTTATAACCGCATGTGCATAC-3′ and 5′-CAGATTTCCCCTTCCTGGTTT-3′ for pri-miR-155; 5′-GCAAGTCGAGCATTTTACCTGC-3′ and 5′-GCCATGTGTCCACTGAAATGTG-3′ for pri-miR-107; 5′-CGGTGCCTACTGAGCTGAT-3′ and 5′-CCTCGGGCACTTACAGACAC-3′ for miR-24; 5′-GCAATTACAGTATTTTAAGAGATGAT-3′ and 5′-CATACTCTACAGTTGTGTTTTAATGT-3′ for pri-miR-16–1.

### RNA-immunoprecipitation and qRT-PCR

Cells were lysed in lysis buffer containing 20 mmol/L HEPES pH 7.9, 150 mmol/L NaCl, 1 mmol/L MgCl_2_, 1 mmol/L Ethylene glycol-bis(β-aminoethyl ether)-N,N,N′,N′-tetraacetic acid (EGTA), 10% glycerol, 1% Triton-X100, 0.1% sodium deoxycholate in the presence of EDTA-free complete protease inhibitor cocktail (Roche), and 20 U/mL RNAse inhibitor (Invitrogen) for 45 minutes, briefly sonicated, and centrifuged at 15,000 × *g* for 15 minutes at 4°C. The supernatants were precleared with protein G beads for 30 minutes, followed by incubation with anti-Flag (M2)-conjugated beads (Sigma) or control IgG-coated beads for 4 hours at 4°C. After wash with lysis buffer for four times, the beads were suspended in 100 μL NT2 buffer (50 mmol/L Tris-HCl pH 7.4, 150 mmol/L NaCl, 1 mmol/L MgCl_2_, 0.05% NP-40) containing 10U DNAase and incubated at 37°C for 10 minutes. RNA–protein complexes were eluted with elution buffer (10 mmol/L Tris-HCl pH8.0, 1 mmol/L EDTA, 1%SDS, 20 U/mL RNAse inhibitor) at 65°C for 10 minutes. The elutes were incubated with Proteinase K (1 mg/mL) at 50°C for 5 hours. RNAs were then extracted using TRIzol reagent (Life Technologies) and subjected to reverse transcription (RT) and quantitative real-time PCR as described above (30). All reactions were carried out in triplicate. Relative gene expression was calculated using the ΔCτ method following the manufacturer's instruction.

### Generation of Tet-inducible DGCR8 Expression Cell Lines

To generate Tet-inducible expression of Flag-DGCR8, HeLa cells were first transfected with pcDNA6-TR (Life Technologies) followed by selection in culture medium containing 5 μg/mL blasticidin to generate HeLa cells stably expressing TR (HeLa-TR). HeLa-TR cells were then transfected with pcDNA4-TO-Flag-DGCR8^WT^ or pcDNA4-TO-Flag-DGCR8^3KR^ and selected in medium containing 5 μg/mL of blasticidin and 100 μg/mL of Zeocin for up to 2 weeks. Single colonies were isolated, expanded, and screened by IB analysis for doxycycline (Dox, 2 μg/mL)-induced expression of DGCR8 using anti-Flag antibody for stable cell lines (HeLa-TO-Flag-DGCR8^WT^ and HeLa-TO-Flag-DGCR8^3KR^). All the cells were cultured in DMEM supplemented with 10% tetracycline system-approved FBS.

### Cell Fractionation

Nucleolar fractionation was performed as described previously (28). Briefly, freshly harvested cells were washed with PBS, resuspended in hypotonic buffer A (10 mmol/L HEPES pH7.8, 10 mmol/L KCl, 1.5 mmol/L MgCl_2_, 0.5 mmol/L DTT) in the presence of PMSF, and incubated for 10 minutes on ice. The cells were homogenized using B pestle douncer followed by spinning down at 3,000 rpm for 5 minutes at 4°C. The supernatant (cytoplasmic fraction) was supplemented with 1/10 volume of buffer B (0.3 mol/L Tris-HCl pH 7.8, 1.4 mol/L KCl, 30 mmol/L MgCl_2_). The nuclear pellets were washed with buffer A and then resuspended in buffer S1 (0.25 mol/L sucrose, 10 mmol/L MgCl_2_), layered over buffer S2 (0.35 mol/L sucrose, 0.5 mmol/L MgCl_2_), and centrifuged at 1,430 × *g* for 10 minutes at 4°C. The pelleted nuclei were resuspended in buffer S2 with PMSF, and sonicated using a microtip probe at power setting at 50%. The sonicated nuclei were then layered over buffer S3 containing 0.88 mol/L sucrose and 0.5 mmol/L MgCl_2_, and centrifuged at 3,000 × *g* for 10 minutes at 4°C. The pellet contained purified nucleoli and the supernatant represented the nucleoplasm.

### Immunofluorescence Staining

Cells were fixed and stained with anti-Flag, anti-NS, anti-Nop58, and anti-USP36 antibodies as indicated, followed by staining with Alexa Fluor 488 (green) goat anti-mouse, Alexa Fluor 546 (red) goat anti-mouse, Alexa Fluor 488 (green) goat anti-rabbit, or Alexa Fluor 546 (red) goat anti-rabbit antibodies (Invitrogen) as well as DAPI for DNA staining. Stained cells were analyzed under a fluorescence microscope (Apotome, Zeiss).

### Cell Proliferation Assay

Cell viability was measured by 3-(4,5-dimethylthiazol-2-yl)-2,5-diphenyltetrazolium bromide (MTT) assays. Briefly, cells were incubated with 0.5 mg/mL MTT in medium for 3 hours. After incubation, MTT medium was removed and DMSO (100 μL per well) was added for fully dissolving the purple formazan. The absorbance was measured at OD560nm and OD690nm. The reduced Abs (Abs560nm-Abs690nm) represents the relative number of viable cells per well. For colony formation assays, equal number of cells were cultured in DMEM containing 10% FBS in the absence or presence of Dox for up to 2 weeks. The colonies were visualized by staining with 0.5% crystal violet in 50% ethanol.

### Data Availability Statement

The data generated in this study are available within the article and its Supplementary Data. Raw data generated in this study are available upon request from the corresponding author.

## Results

### Knockdown of USP36 Attenuates miRNA Biogenesis

**FIGURE 1:**
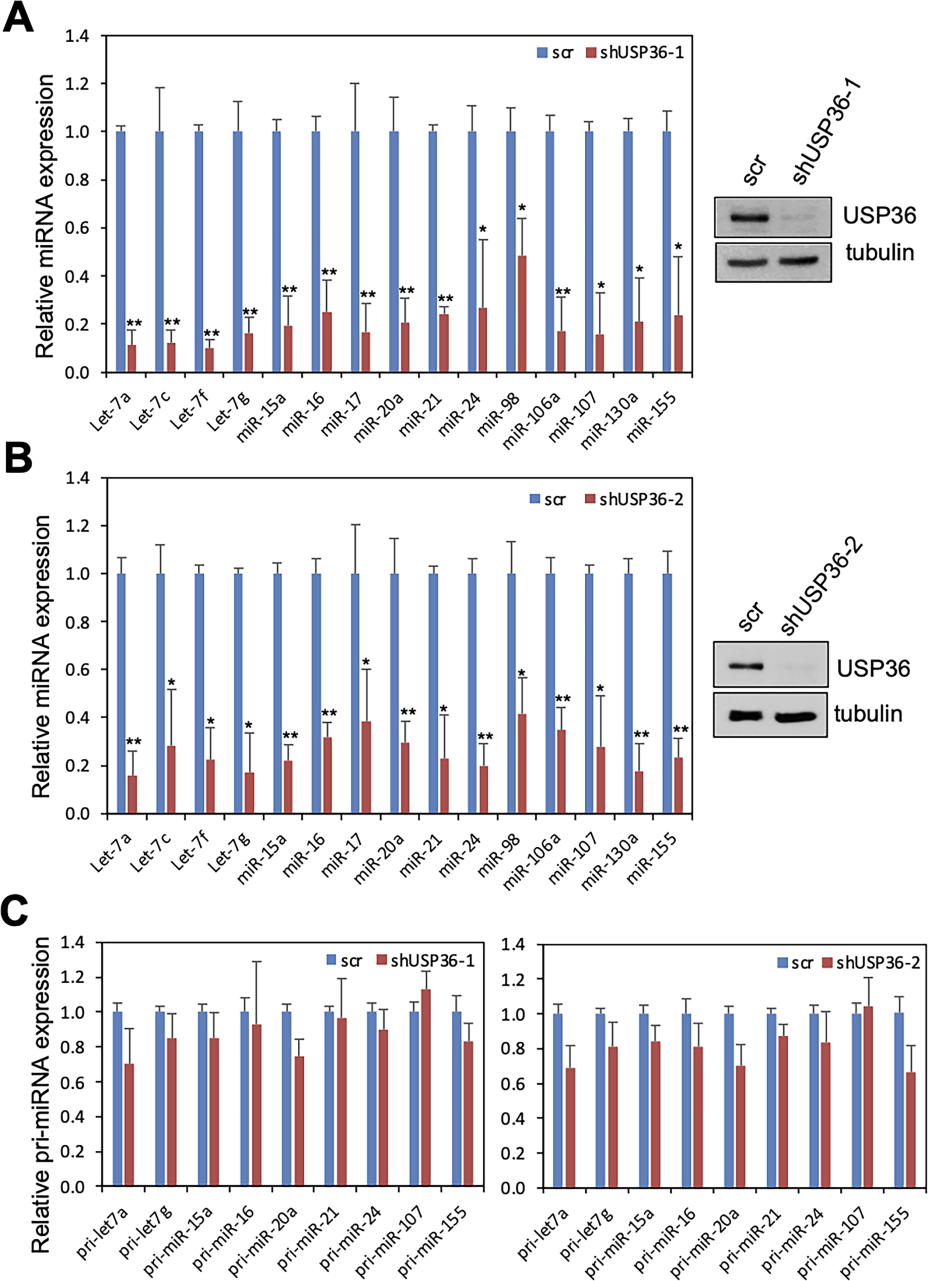
Knockdown of USP36 reduces the levels of mature miRNAs, but not pri-miRNAs. **A** and **B,** Expression of mature miRNAs upon USP36 knockdown. H1299 cells were infected with scrambled (scr) or USP36 shRNA-1 (**A**) or shRNA-2 (**B**) lentiviruses followed by qRT-PCR detection of the indicated miRNAs. Shown are the relative fold changes of miRNA levels normalized to U6 RNA as an internal control in USP36 shRNA infected cells compared with scr infected cells. Data were presented as mean ± SD of three independent experiments. *, *P* < 0.05; **, *P* < 0.01, compared with scr control as determined by Student *t* test. The expression of USP36 assayed by IB is shown in the right. **C,** The expression of pri-miRNAs upon USP36 knockdown. Above H1299 cells infected with scr or USP36 shRNA lentiviruses were also assayed by qRT-PCR to detect the indicated pri-miRNAs. Shown are the relative fold changes of pri-miRNA levels normalized to U6 RNA as an internal control in USP36 shRNA infected cells compared with scr infected cells.

USP36 plays an important role in ribosome biogenesis including rRNA processing (24–28). A recent study using miRNA screening of RBPs showed that USP36 is able to bind to several miRNA precursors (32). To understand whether USP36 plays a role in miRNA biogenesis, we first performed USP36 knockdown experiments to determine whether depletion of USP36 affects the levels of mature miRNAs. As shown in Fig. 1A, knockdown of USP36 by lentiviral encoded shRNA markedly reduced the levels of all tested mature miRNAs compared with scrambled control in H1299 cells. Similar results were also observed in cells infected with a different USP36 shRNA lentivirus (Fig. 1B). Also, knockdown of USP36 reduced the levels of tested mature miRNAs in HeLa cells (Supplementary Fig. S1A) as well as human immortalized normal fibroblast IMR-90 cells (Supplementary Fig. S1B). Interestingly, knockdown of USP36 does not significantly reduce the levels of the tested pri-miRNAs in cells (Fig. 1C). These results suggest that USP36 does not significantly affect pri-miRNA transcription, but instead it plays a critical role in miRNA processing.

### USP36 Associates with the Drosha-DGCR8 Microprocessor Complex

**FIGURE 2:**
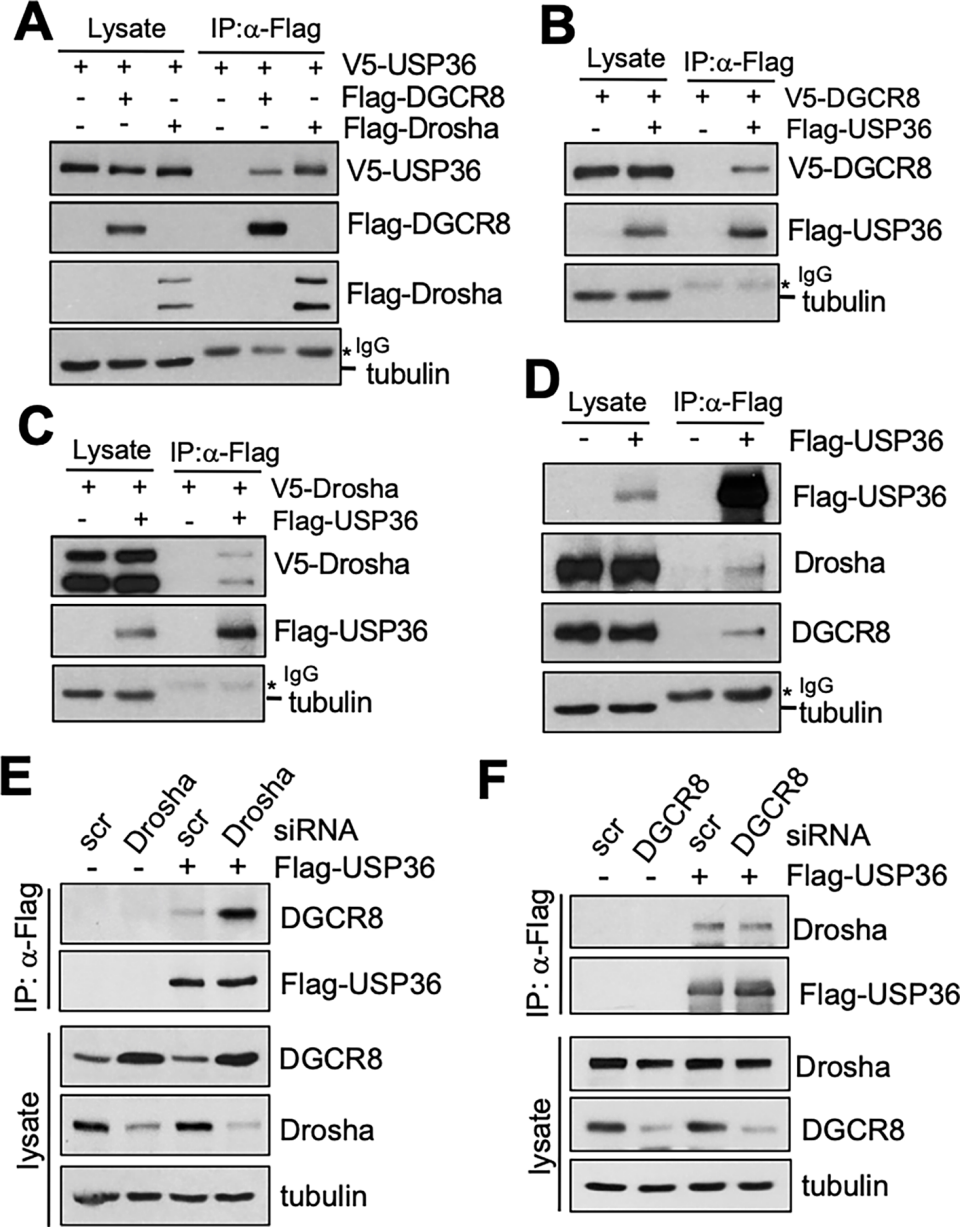
USP36 interacts with DGCR8 and Drosha. **A,** H1299 cells transfected with V5-USP36 without or with Flag-DGCR8 or Flag-Drosha were subjected to co-IP with anti-Flag antibody followed by IB. **B,** H1299 cells transfected with V5-DGCR8 in the absence or presence of Flag-USP36 were subjected to co-IP with anti-Flag antibody followed by IB. **C,** H1299 cells transfected with V5-Drosha in the absence or presence of Flag-USP36 were subjected to co-IP with anti-Flag antibody followed by IB. **D,** co-IP of Flag-USP36 with endogenous DGCR8 and Drosha. H1299 cells transfected with Flag-USP36 or control were assayed by co-IP using anti-Flag antibody followed by IB. **E,** Knockdown of Drosha does not reduce USP36-DGCR8 binding. 293 cells transfected with Flag-USP36 together with scr control or Drosha siRNA were subjected to co-IP using anti-Flag antibody followed by IB. **F,** Knockdown of DGCR8 does not reduce USP36-Drosha binding. 293 cells transfected with Flag-USP36 together with scr control or DGCR8 siRNA were subjected to co-IP using anti-Flag antibody followed by IB.

USP36 is primarily a nucleolar protein (24, 28, 33) and it has been shown that the Drosha-DGCR8 microprocessor complex is nuclear proteins that can be located in the nucleolus (34). Indeed, we observed that exogenously expressed Flag-DGCR8 and GFP-DGCR8 are predominantly localized in the nucleolus, whereas endogenous DGCR8 localized in both the nucleoplasm and nucleolus (Supplementary Fig. S2A). DGCR8 colocalized with both exogenously expressed USP36 and endogenous USP36 in the nucleolus (Supplementary Fig. S2B). Cell fractionation assays further confirmed that a substantial portion of endogenous DGCR8 and Drosha are present in the nucleolar fraction (Supplementary Fig. S2C). Thus, we reasoned that USP36 may regulate primary miRNA processing mediated by microprocessor complex, likely in the nucleolus. We therefore tested whether USP36 interacts with the Drosha-DGCR8 microprocessor complex. Indeed, exogenously expressed V5-USP36 was co-immunoprecipitated with Flag-DGCR8 and Flag-Drosha (Fig. 2A) and Flag-USP36 co-immunoprecipitates with V5-DGCR8 (Fig. 2B) and V5-Drosha (Fig. 2C) by using anti-Flag antibody in cells. Also, endogenous DGCR8 and Drosha were immunoprecipitated with Flag-USP36 using anti-Flag antibody (Fig. 2D). Together, the results suggest that USP36 interacts with the Drosha-DGCR8 microprocessor complex in cells.

To examine whether USP36 can interact with DGCR8 and Drosha independently, we knocked down DGCR8 or Drosha and tested their reciprocal interaction with USP36. As shown in Fig. 2E, knockdown of Drosha did not abolish the interaction of USP36 with DGCR8. Instead, knockdown of Drosha drastically increased the levels of DGCR8, due to the posttranscriptional inhibition mechanism of DGCR8 by Drosha as reported previously (35), and therefore increased the USP36-DRCR8 interaction. Similar results were also observed using a Drosha siRNA pool (Supplementary Fig. S3A). Knockdown of DGCR8 also did not reduce the interaction of USP36 with Drosha (Fig. 2F; Supplementary Fig. S3B). Altogether, our results suggest that USP36 forms a complex with DGCR8-Drosha by interacting with both DGCR8 and Drosha.

### Both the N-terminus and C-terminus Domains of USP36 are Involved in the Interaction with Drosha and DGCR8

**FIGURE 3:**
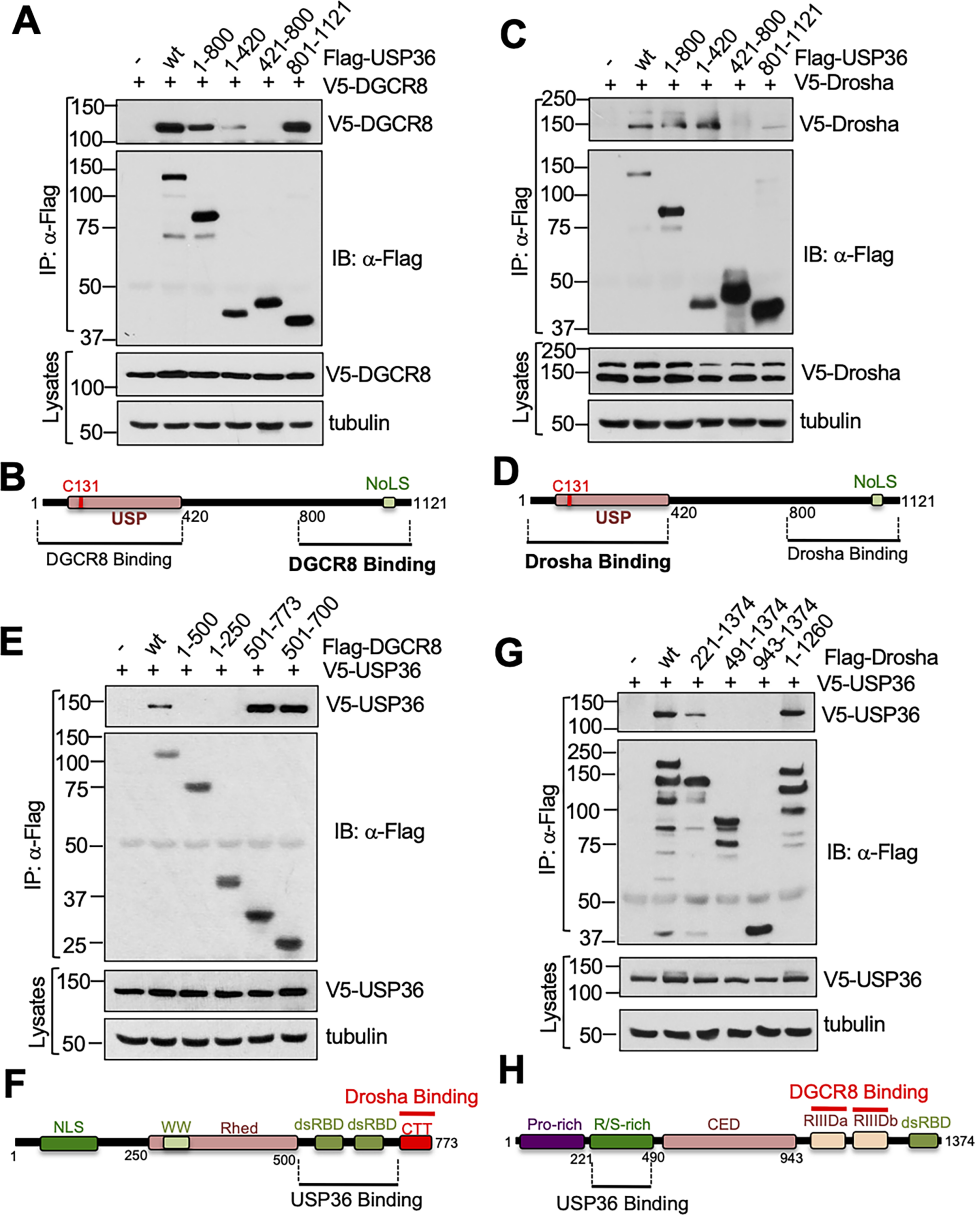
Mapping the binding of USP36 to DGCR8 and Drosha. **A** and **B,** DGCR8 binds to both the N-terminal and C-terminal domains of USP36. H1299 cells were transfected with V5-DGCR8 together with Flag-USP36 or its deletion mutants as indicated, followed by co-IP with anti-Flag and IB analysis (**A**). The diagram of DGCR8 binding to USP36 domains is shown in **B**. USP: ubiquitin-specific protease; NoLS, nucleolar localization signal. **C** and **D,** Drosha binds to both the N-terminal and C-terminal domains of USP36. H1299 cells were transfected with V5-Drosha together with Flag-USP36 or its deletion mutants as indicated, followed by co-IP with anti-Flag and IB analysis (**C**). The diagram of Drosha binding domains of USP36 is shown in **D**. **E** and **F,** USP36 binds to the C-terminus of DGCR8. H1299 cells were transfected with V5-USP36 together with full-length Flag-DGCR8 or its deletion mutants. Cell lysates were immunoprecipitated with anti-Flag antibody followed by IB. The diagram of USP36 binding to the C-terminus of DGCR8 is shown in **F**. **G** and **H,** USP36 binds to the R/S-rich domain of Drosha. H1299 cells were transfected with V5-USP36 together with full-length Flag-Drosha or its deletion mutants followed by co-IP with anti-Flag antibody and IB. The diagram of USP36 binding to the R/S-rich domain of Drosha is shown in **H**.

To understand how USP36 interacts with the microprocessor complex, we examined which USP36 domain(s) bind to Drosha and DGCR8 using co-IP IB assays. We transfected cells with V5-DGCR8 together with control, Flag-tagged full-length USP36, and a panel of Flag-USP36 deletion mutants followed by co-IP using anti-Flag antibody. As shown in Fig. 3A and summarized in Fig. 3B, both the N-terminal USP domain containing (amino acids 1–420) and the C-terminal nucleolar localization signal (24, 33) containing region (aa 801–1121), but not and the middle (aa 421–800) region, interacts with DGCR8 with the C-terminus binding stronger than the N-terminus (compare lane 6 with lane 4; Fig. 3A). Similarly, co-IP assays using lysates from cells transfected with V5-Drosha in the absence or presence of Flag-USP36 or its deletion mutants showed that Drosha also interacts with both the N-terminus and the C-terminus of USP36 (Fig. 3C and D). Interestingly, the N-terminus of USP36 binds to Drosha stronger than the C-terminus (compare lane 6 with lane 4; Fig. 3C). These results further suggest that USP36 may form multiprotein complex with Drosha and DGCR8 with major contact for Drosha and DGCR8 being at the N-terminus and C-terminus of USP36, respectively (Fig. 3B–D).

To examine where USP36 binds to Drosha and DGCR8, we also performed co-IP and IB experiments using a panel of Flag-DGCR8 mutants (Fig. 3E and F) and Flag-Drosha mutants (Fig. 3G and H). As show in Fig. 3E, USP36 interacts with the C-terminus region of DGCR8 that contains two dsRBD domains. We also mapped that the N-terminal Arginine-Serine rich (R/S-rich) domain of Drosha, a region different from the C-terminal two RIIID domains where two DGCR8 molecules bind (10, 11), is necessary for USP36 interaction (Fig. 3G and H). Given that recent structural studies have shown that the C-terminal CTT domain of two DGCR8 bind to Drosha (10, 11), above results suggest that USP36 may simultaneously interact with both DGCR8 and Drosha, forming a multiprotein complex.

### USP36 does not Significantly Affect the Levels of DGCR8 and Drosha

**FIGURE 4:**
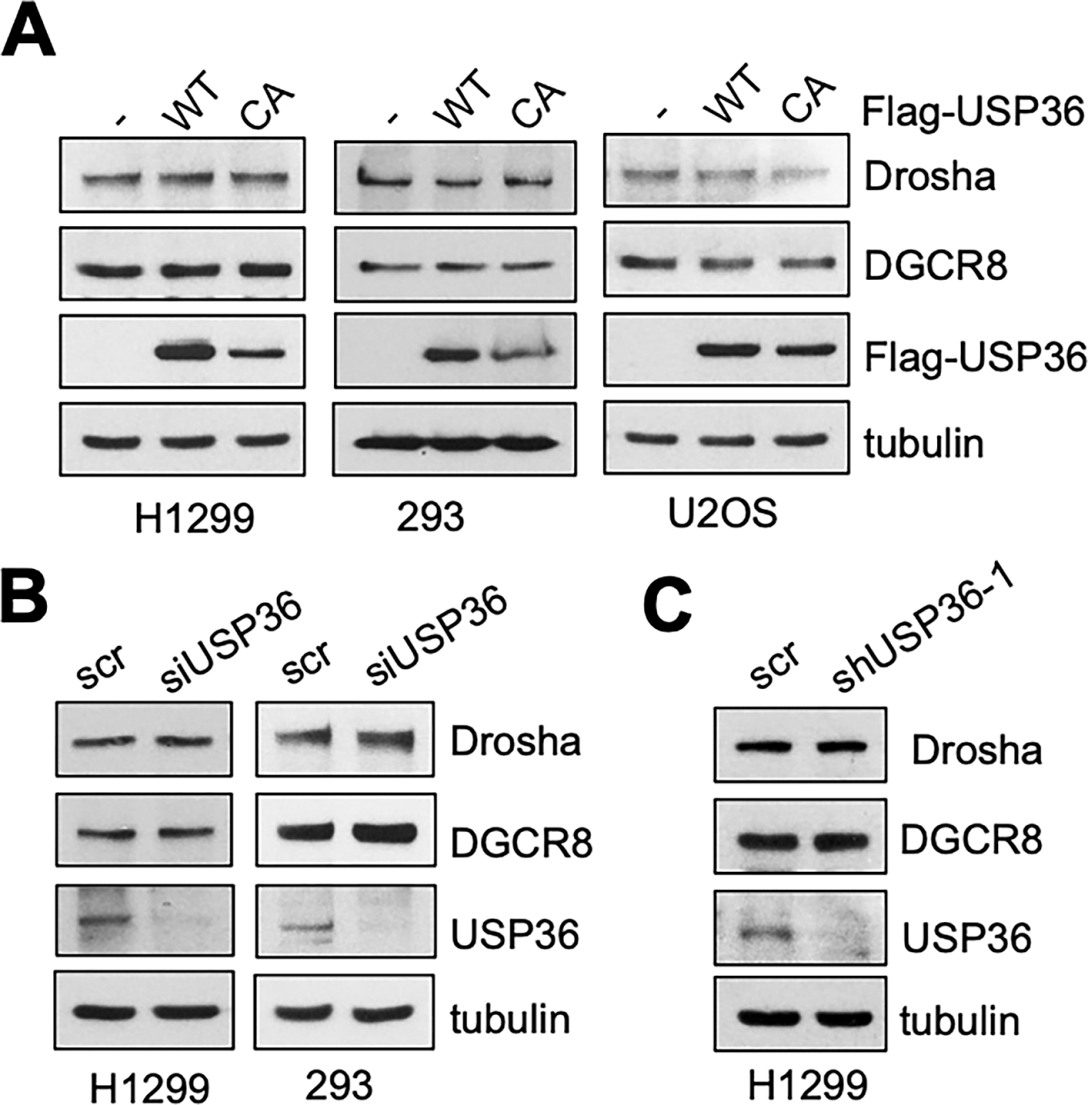
USP36 does not affect the levels of DGCR8 and Drosha. **A,** Overexpression of USP36 does not increase the levels of DGCR8 and Drosha. H1299, 293, or U2OS cells were transfected with WT USP36 or its catalytically-inactive C131A (CA) mutant and assayed for protein expression by IB. Knockdown of USP36 does not reduce the levels of DGCR8 and Drosha. H1299 and 293 cells transfected with scr or USP36 siRNA (**B**) and H1299 cells infected with scr or USP36 shRNA (**C**) were assayed by IB.

As USP36 is a nucleolar deubiquitinating enzyme (DUB; refs. 24, 28), we examined whether USP36 regulates the levels of DGCR8 and Drosha. As shown in Fig. 4A, overexpression of USP36 did not increase the levels of DGCR8 and Drosha in multiple tested cell lines. Also, knockdown of USP36 by siRNA (Fig. 4B) or lentiviral-encoded shRNA (Fig. 4C) did not reduce the levels of DGCR8 and Drosha. These results indicate that USP36 does not affect the steady-state levels of the microprocessor complex. Indeed, although we did observe the marginal ubiquitination of exogenously expressed DGCR8 that can be deubiquitinated by WT USP36, but not the catalytically-inactive C131A mutant (Supplementary Fig. S4A), the levels of endogenous DGCR8 ubiquitination are below detectable (Supplementary Fig. S4B), suggesting that the steady-state levels of DGCR8 ubiquitination under normal cell growth conditions are too low to be regulated by USP36.

### USP36 SUMOylates DGCR8

**FIGURE 5:**
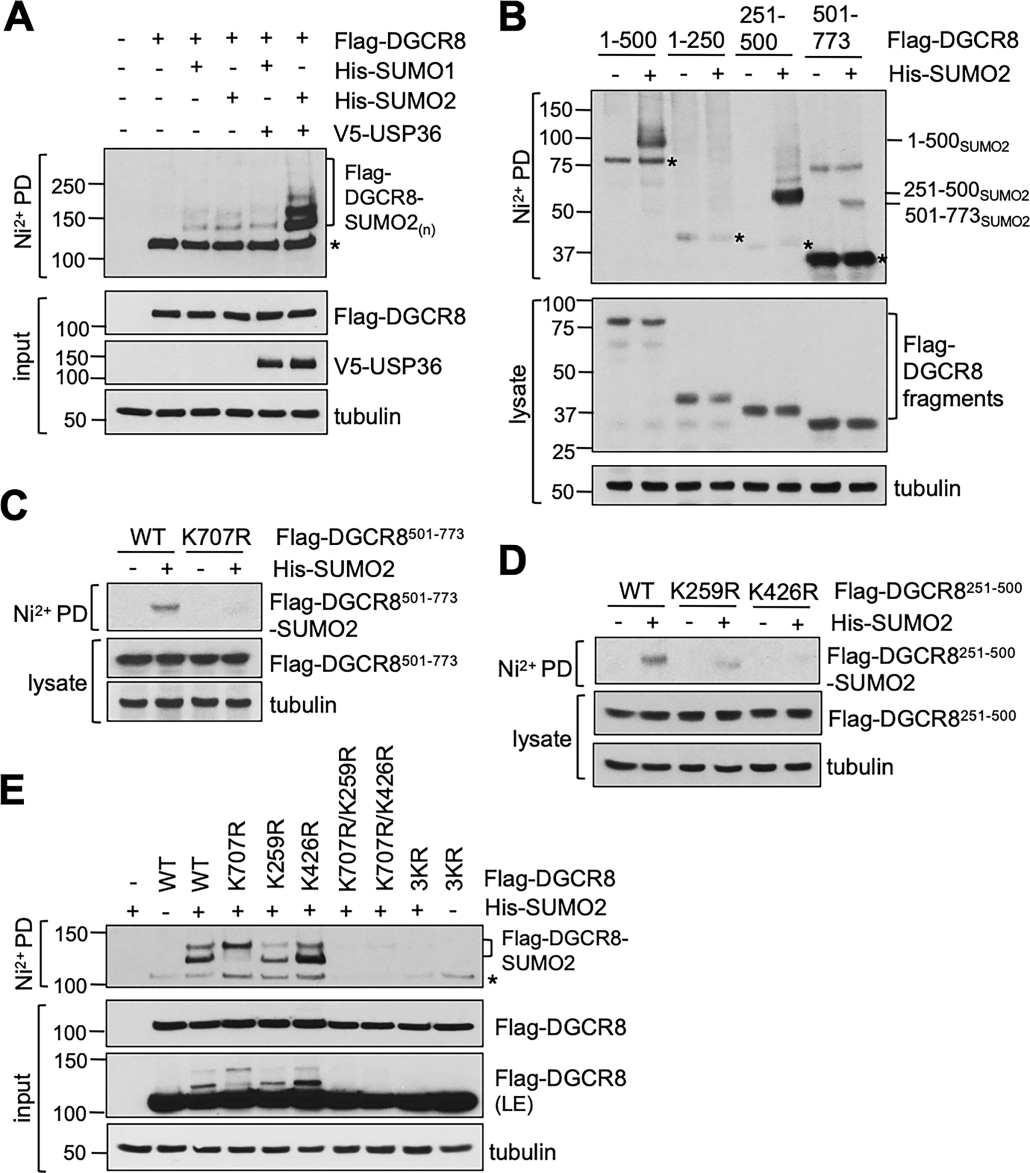
USP36 promotes DGCR8 SUMOylation. **A,** USP36 promotes DGCR8 SUMOylation by SUMO2. H1299 cells transfected with the indicated plasmids were subjected to Ni^2+^-NTA PD under denaturing conditions, followed by IB with anti-Flag antibody to detect DGCR8 SUMOylation. The protein expression is shown in bottom. * indicates unmodified DGCR8. **B,** SUMOylation of DGCR8 deletion mutants. H1299 cells were transfected with the individual DGCR8 deletion mutants without or with His-SUMO2, followed by Ni^2+^-NTA PD under denaturing conditions to detect the SUMOylation of DGCR8 fragments. The protein expression is shown in the bottom. * indicates unmodified DGCR8 fragments. **C,** Mutating K707 to R abolishes the SUMOylation of the C-terminus fragment of DGCR8. H1299 cells were transfected with the indicated plasmids and assayed for SUMOylation by Ni^2+^-NTA PD under denaturing conditions, followed by IB. **D,** Mutating K259 or K426 attenuated the SUMOylation of the central fragment of DGCR8. H1299 cells were transfected with the indicated plasmids and assayed for SUMOylation by Ni^2+^-NTA PD, followed by IB. **E,** Characterization of SUMO acceptor sites at DGCR8. H1299 cells transfected with His-SUMO2 together with WT Flag-DGCR8 or the indicated mutant plasmids were subjected to Ni^2+^-NTA beads PD under denaturing conditions followed by IB. 3KR indicates the Flag-DGCR8^K259R/K426R/K707R^ mutant. * indicates unmodified DGCR8.

We have recently shown that USP36 also possesses a SUMO ligases activity and promotes nucleolar protein group SUMOylation (27). DGCR8 has been previously shown to be subjected to SUMO modification (23, 36). Therefore, we examined whether USP36 regulates DGCR8 via SUMOylation. We transfected cells with Flag-DGCR8 in the absence or presence of USP36, SUMO1, or SUMO2 and performed *in vivo* SUMOylation assays under denaturing conditions using Ni^2+^-NTA PD methods. As shown in Fig. 5A, DGCR8 can be modified by both SUMO1 and SUMO2. Intriguingly, coexpression of USP36 markedly increased the SUMOylation of DGCR8 modified by SUMO2, but not SUMO1. The effect of USP36 in promoting DGCR8 SUMOylation does not depend on its DUB activity, as the DUB catalytically inactive but SUMO ligase active mutant as we have described previously (27), H382A (the DUB catalytic protein acceptor His 382 is mutated to Ala), promoted DGCR8 SUMOylation as efficiently as WT USP36 (Supplementary Fig. S4C). There are several putative SUMOylation sites located in both the middle and the C-terminal region of DGCR8 and lysine (Lys, K) 707 and K259 have been shown to be among SUMO acceptors (23, 36). To examine which Lys residues in DGCR8 are subjected to SUMO2 modification, we performed *in vivo* SUMOylation assays using the Flag-DGCR8 deletion mutants and showed that both the central (aa 251–500) and the C-terminal (aa 501–773) regions can be SUMOylated by SUMO2 (Fig. 5B). Mutating K707 to Arginine (Arg; K707R) indeed abolished the SUMOylation of the C-terminus region (Fig. 5C), but not full-length DGCR8 (Fig. 5E). Also, mutating K259 to Arg (K259R) did not completely abolish the SUMOylation of the middle region and the full-length DGCR8 (Fig. 5D and E). We also examined another putative canonical SUMO site at K426 and found that mutating K426 to Arg (K426R) also markedly reduced the SUMOylation of the middle region but not the full-length DGCR8 (Fig. 5D and E). We therefore created double and triple Lys to Arginine (Arg) mutants and performed *in vivo* SUMOylation assays as shown in Fig. 5E. Interestingly, mutating both K259 and K707 eliminates majority of DGCR8 SUMOylation and so does for mutating K707 and K426. Mutating all the three Lys residues completely abolished DGCR8 SUMOylation. These results suggest that all these three Lys residues are SUMO sites for DGCR8 and K707 and K259 are the two main SUMO acceptors.

### DGCR8 SUMOylation Promotes its Targeting of pri-miRNAs

**FIGURE 6:**
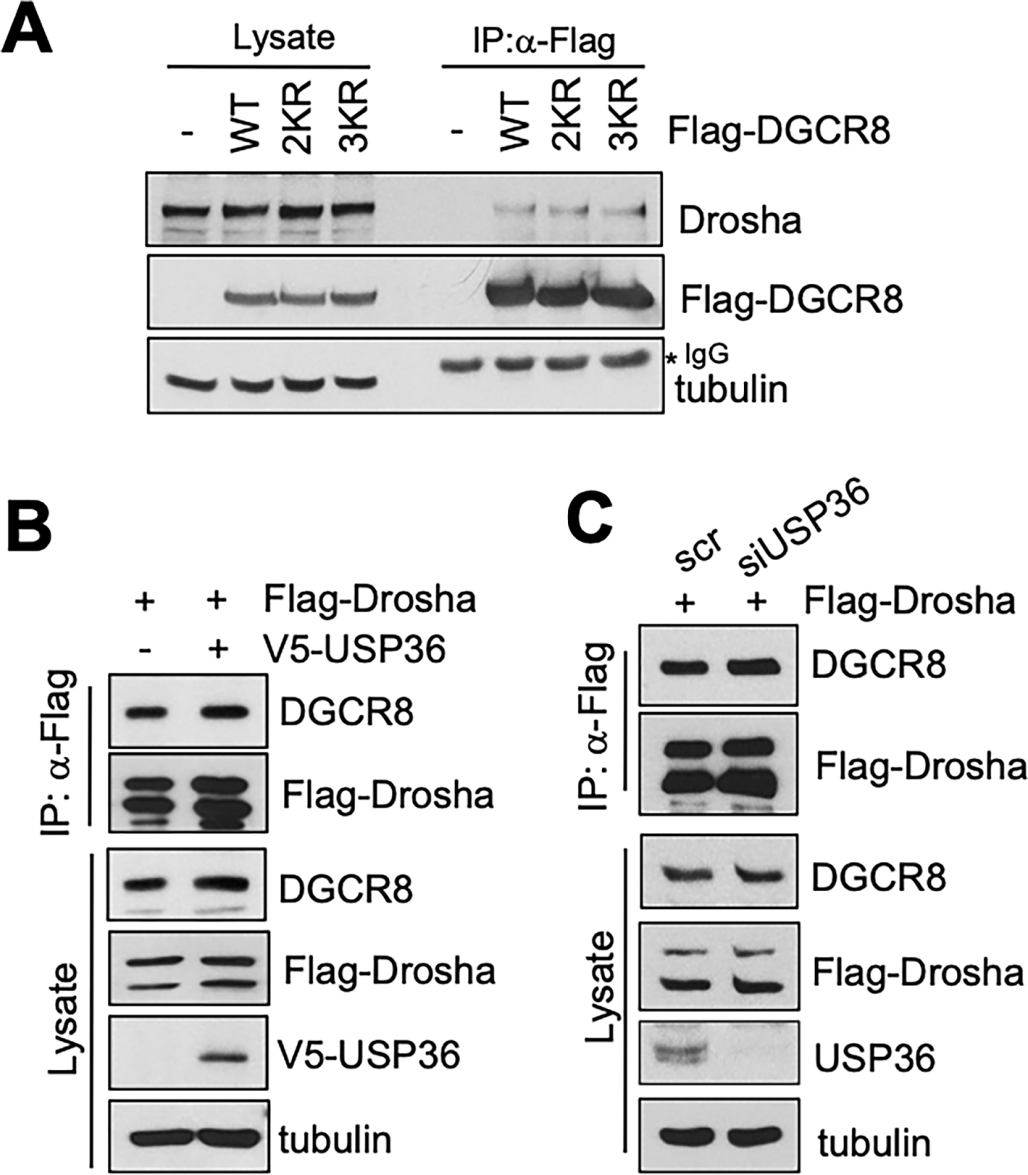
SUMOylation of DGCR8 by USP36 does not affect the microprocessor complex formation. **A,** Abolishing DGCR8 SUMOylation does not affect its binding to Drosha. 293 cells transfected with WT Flag-DGCR8 or its SUMOylation-defective mutants were subjected to co-IP with anti-Flag antibody followed by IB. 2KR and 3KR indicates the Flag-DGCR8^K259R/K707R^ and Flag-DGCR8^K259R/K426R/K707R^ mutants, respectively. **B,** Overexpression of USP36 does not promote the binding of DGCR8 with Drosha. 293 cells transfected with Flag-Drosha in the absence or presence of V5-USP36 were assayed by co-IP using anti-Flag followed by IB. **C,** Knockdown of USP36 does not reduce the binding of DGCR8 with Drosha. 293 cells transfected with Flag-Drosha in the presence of scr or USP36 siRNA were assayed by co-IP using anti-Flag followed by IB.

To understand the function of DGCR8 SUMOylation in regulating the microprocessor complex, we first tested whether abolishing DGCR8 SUMOylation affects the level of DGCR8 and the microprocessor complex formation. As shown in Fig. 6A, neither K259 and K707 double mutant nor K259, K426, and K707 triple mutant affected the levels of DGCR8 or its interaction with Drosha. Furthermore, overexpression of USP36 did not significantly increase the binding of Drosha with DGCR8 (Fig. 6B) and knockdown of USP36 did not reduce the Drosha interaction with DGCR8 (Fig. 6C), suggesting that USP36-mediated DGCR8 SUMOylation does not affect the microprocessor complex formation.

**FIGURE 7:**
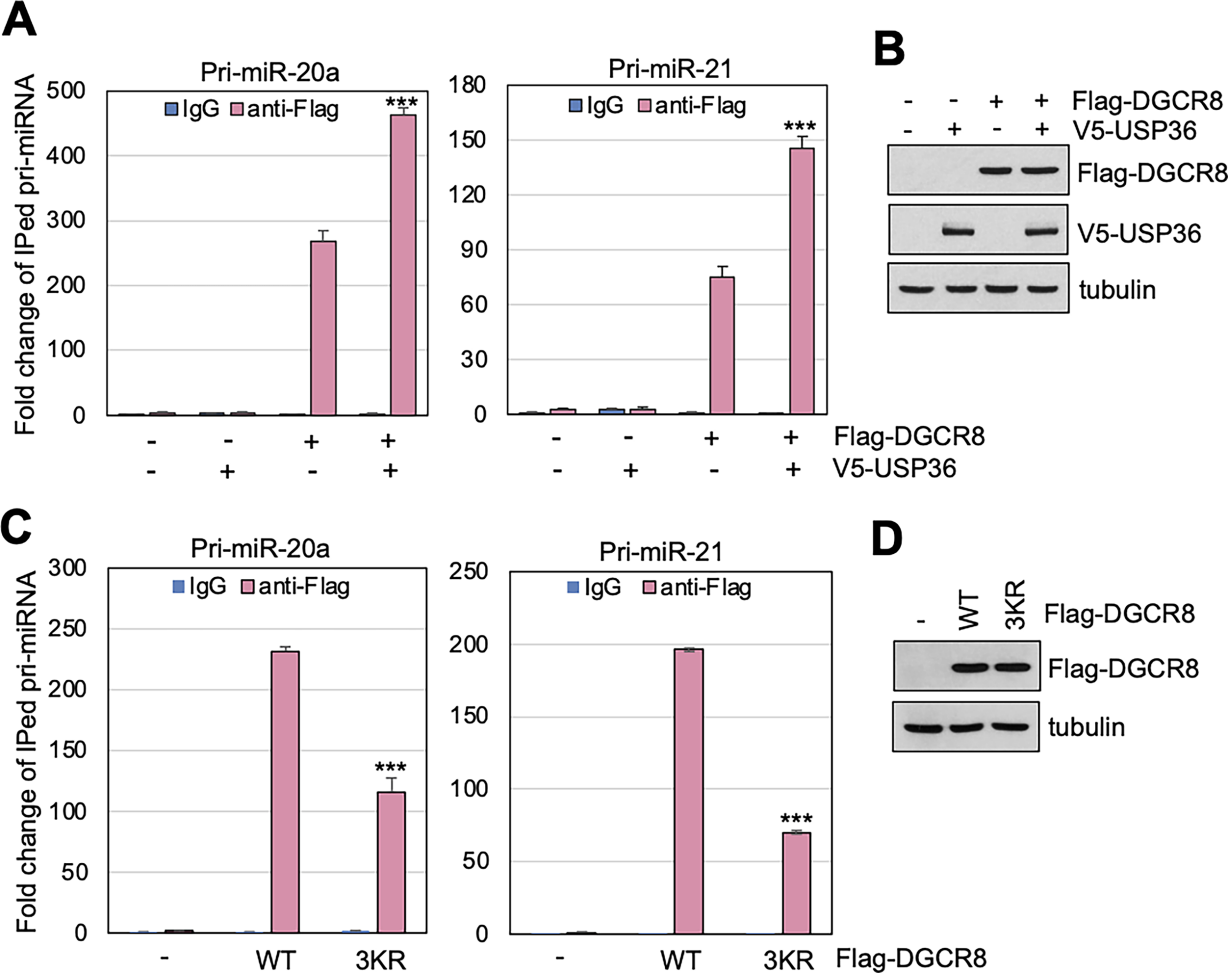
The SUMOylation of DGCR8 is critical for its binding to pri-miRNA. **A** and **B,** Overexpression of USP36 promotes DGCR8 binding to the tested pri-miRNAs**.** H1299 cells transfected with Flag-DGCR8 together with or without V5-USP36 were subjected to RNA-IP with anti-Flag, followed by qRT-PCR detection of the indicated pri-miRNAs. Shown are fold changes of immunoprecipitated pri-miRNA, determined by comparing IgG control in empty vector transfected cells normalized to input, in one representative experiment from three independent experiments (**A**). Data were presented as mean ± SD of three technical replicates. *P* values shown were calculated by Student *t* test. ***, *P* < 0.001. The expression of Flag-DGCR8 and V5-USP36 assayed by IB is shown in **B**. **C** and **D,** Abolishing DGCR8 SUMOylation impairs its binding to the tested pri-miRNAs. H1299 cells transfected with control or Flag-DGCR8^3KR^ mutant plasmid were subjected to RNA-IP with anti-Flag followed by qRT-PCR analysis. Shown are fold changes of immunoprecipitated pri-miRNAs, determined by comparing IgG control in empty vector transfected cells normalized to input, in one representative experiment from three independent experiments (**C**). Data were presented as mean ± SD of three technical replicates. *P* values shown were calculated by Student *t* test. ***, *P* < 0.001. The expression of Flag-DGCR8 proteins assayed by IB was shown in **D**.

We then examined whether USP36-mediated SUMOylation of DGCR8 affects its ability to bind to its targeting pri-miRNAs. To do so, we transfected cells with DGCR8 in the presence or absence of USP36, followed by RNA-immunoprecipitation (RNA-IP) and qRT-PCR to detect DGCR8 binding to pri-miRNAs. As shown in Fig. 7A and B, overexpression of USP36 markedly increased the binding of DGCR8 to the tested pri-miRNAs such as pri-miR-20a and pri-miR-21. Also, abolishing DGCR8 SUMOylation by mutating K259, K426, and K707 to residues Arg (3KR) significantly attenuated the binding of DGCR8 to the tested pri-miR-20a and pri-miR-21 (Fig. 7C and D). Therefore, our results suggest that SUMOylation of DGCR8 by USP36 promotes DGCR8 association with pri-miRNA and thus the pri-miRNA processing.

### Ablation of DGCR8 SUMOylation Inhibits Cell Growth

**FIGURE 8:**
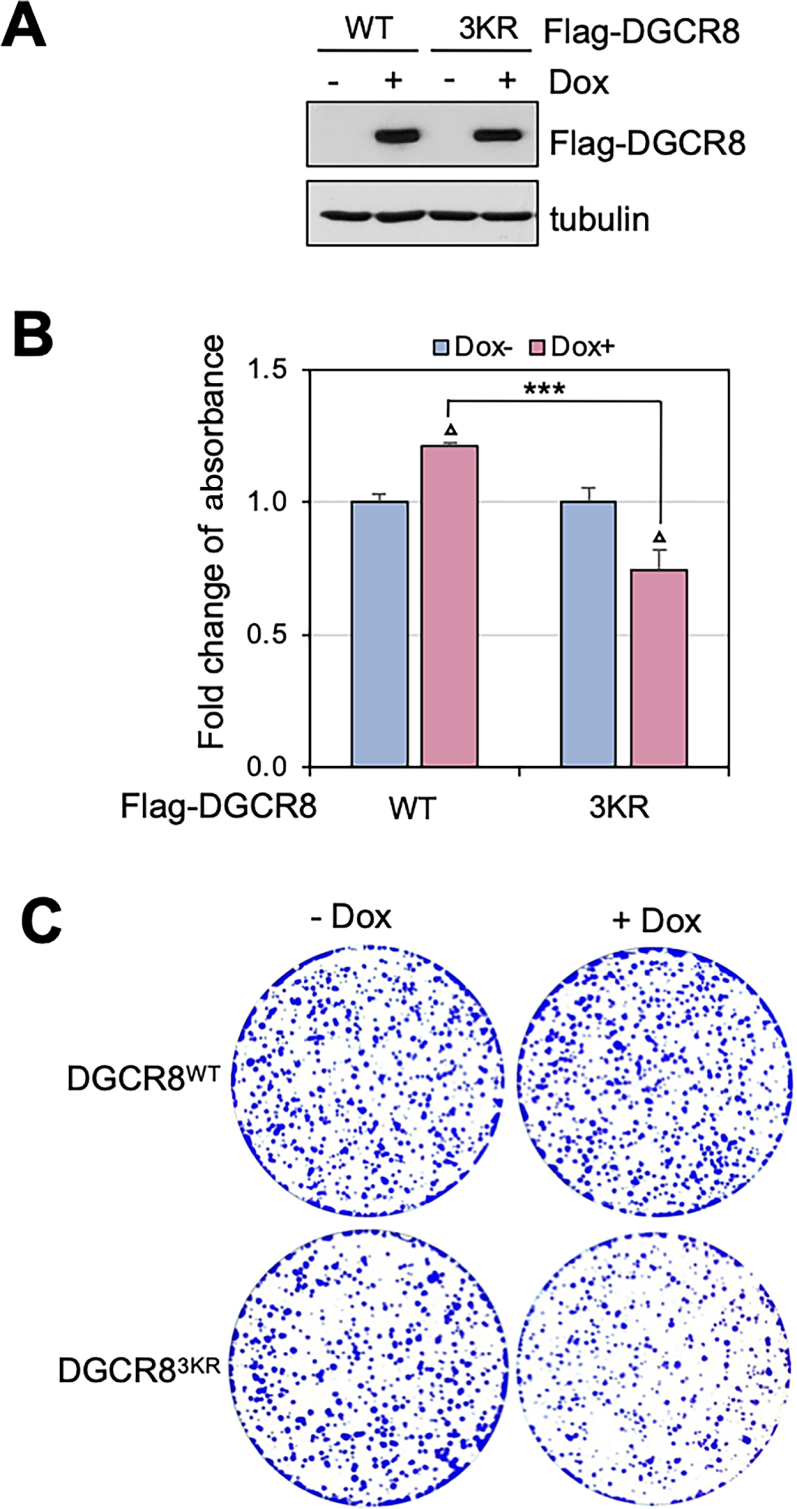
The SUMOylation of DGCR8 is critical for cell proliferation. **A,** Establishment of tet-inducible expression of DGCR8. HeLa-TO-Flag-DGCR8^WT^ or HeLa-TO-Flag-DGCR8^3KR^ cell lines were incubated in the absence or presence of 2 μg/mL Dox for 24 hours, followed by IB. **B,** Induced expression of Flag-DGCR8^3KR^ suppresses cell growth. Above HeLa-TO-Flag-DGCR8^WT^ and HeLa-TO-Flag-DGCR8^3KR^ cells were cultured in the absence or presence of 2 μg/mL Dox for 96 hours followed by MTT assays (**B**). Shown are the fold changes of absorbance from three independent experiments. Data were presented as mean ± SD. *P* values shown were calculated by Student *t* test. Δ*P* < 0.05, compared with cells culture in the absence of Dox. ***, *P* < 0.001, compared HeLa-TO-Flag-DGCR8^WT^ cells with HeLa-TO-Flag-DGCR8^3KR^ cells cultured in the presence of Dox. **C,** HeLa-TO-Flag-DGCR8^WT^ and HeLa-TO-Flag-DGCR8^3KR^ cells were cultured in the absence or presence of Dox for colony formation assays. Shown is one representative colony formation from three independent experiments.

To understand the biological function of DGCR8 SUMOylation by USP36, we established the tet-inducible expression of WT DGCR8 and the SUMOylation-defective DGCR8^3KR^ mutant in HeLa cells (Fig. 8A) and perform cell proliferation assays. As shown in Fig. 8B, while Dox-induced expression of WT DGCR8 slightly increased cell viability and proliferation, expression of the 3KR mutant significantly suppressed cell viability and proliferation, as measured by MTT assays. Colony formation assays also showed that Dox-induced expression of DGCR8^3KR^ markedly inhibited cell proliferation (Fig. 8C). Together, these results suggest that the SUMOylation-defective DGCR8^3KR^ acts as a dominant-negative mutant suppressing endogenous DGCR8 function and thus, SUMOylation of DGCR8 is critical for its function in cell growth and proliferation.

## Discussion

In this study, we report that USP36 regulates miRNA biogenesis by SUMOylating DGCR8. We show that USP36 interacts with the DGCR8-Drosha microprocessor complex. Interestingly, USP36, as a DUB enzyme, does not affect the ubiquitination and turnover of DGCR8. Instead, it mediates SUMOylation of DGCR8 and promotes the binding of DGCR8 to pri-miRNAs and their processing. Consequently, USP36 depletion markedly reduces the levels of tested mature miRNAs but not their respective pri-miRNAs. These results identify USP36 as a novel regulator of the microprocessor complex, adding it to the growing list of posttranscriptional and posttranslational regulators of miRNA biogenesis.

The regulation of DGCR8 by USP36 is reminiscent of the regulation of the small nucleolar ribonucleoprotein (snoRNP) complexes where USP36 acts as a SUMO ligase, but not a DUB, to promote snoRNP protein SUMOylation, without affecting their protein levels, and facilitates snoRNP protein binding to snoRNAs (27). Similarly, DGCR8 SUMOylation by USP36 does not affect the DGCR8-Drosha microprocessor complex formation but promotes DGCR8 binding to pri-miRNAs, suggesting that SUMOylation of DGCR8 by USP36 increases its affinity to pri-miRNAs. This is supported by other posttranslational modifications of DGCR8, such as DGCR8 deacetylation that increases its affinity to pri-miRNAs (37). It is likely that SUMOylation of DGCR8 may induce confirmational changes that favor the microprocessor complex targeting of pri-miRNAs and thus increase the activity of Drosha-mediated processing of the pri-miRNAs.

Our binding domain mapping results reveal that USP36 binds to DGCR8 and Drosha via distinct domains, suggesting that the three proteins form a multiprotein complex. Both DGCR8 and Drosha can bind to the N-terminus and C-terminus of USP36 albeit DGCR8 binds strongly to the C-terminus of USP36 whereas Drosha binds strongly to the N-terminus of USP36. Also, USP36 binds to the central R/S-rich region that is distinct from the DGCR8 binding RNaseIII domains at the C-terminus of Drosha. Furthermore, USP36 binds to the dsRBD domains that are distinct from the Drosha binding CTT domain at the C-terminus of DGCR8. Thus, USP36 interacts with microprocessor complex by contacting both DGCR8 and Drosha. Both DGCR8 and Drosha have been shown to localize in the nucleolus (34, 38) and our immunofluorescence and cell fractionation assays also support that DGCR8 and Drosha can localize in the nucleolus (Supplementary Fig. S2). As USP36 is primarily a nucleolar protein (24, 28, 33) and it colocalizes with DGCR8 in the nucleolus (Supplementary Fig. S2B), our results suggest that USP36 may regulate pri-miRNA processing by microprocessor complex via SUMOylating DGCR8 in the nucleolus. It is likely that pri-miRNAs may associate with the microprocessor complex in the nucleoplasm and then translocated into the nucleolus, possibly by binding an anchor protein nucleolin as suggested (34, 39) and USP36 may stabilize the complex and facilitate the miRNA processing activity via SUMOylating DGCR8 in the nucleolus.

Of note, we observed that USP36 specifically promotes DGCR8 SUMOylation by SUMO2, but not SUMO1, although it can promote protein SUMOylate by either SUMO1 or SUMO2 (27). This is intriguing, yet the mechanism underlying this specificity is currently unknown. One possibility is that the above USP36-microprocessor complex creates a conformation that favors the docking of Ubc9-charged SUMO2 but not SUMO1. Future structural studies are warranted to characterize the structure of the USP36-microprocessor complex and its conformational change upon recruiting Ubc9-charged SUMO2. Nevertheless, this SUMO2 specificity further elucidates a fine-tuned regulation of miRNA biogenesis by USP36.

Our functional study showed that DGCR8 SUMOylation is critical for DGCR8 function in cell proliferation (Fig. 8), correlating with the essential role for USP36 in cell growth and proliferation (25, 27, 40). As USP36 is frequently overexpressed in various human cancers (28, 41, 42), its role in miRNA biogenesis may be deregulated in cancer cells, especially those oncogenic miRNAs. For example, miRNAs tested in this study including miR-21, miR-155, miR-17, and miR-20a have been shown upregulated in various human cancers (43, 44). Indeed, deregulation of DGCR8 and Drosha has also been linked to tumorigenesis (9, 45). Future studies may also include the characterization of the USP36-miRNA biogenesis pathway in tumorigenesis and tumor growth. A number of auxiliary factors implicated in cancer, such as p53, SMAD, and KSRP, regulate the processing of subsets of pri-miRNAs by acting on microprocessor complex (16–18). Thus, it will also be interesting to investigate whether USP36 regulates the expression of specific subsets of miRNAs that may control cell growth, cell death, and differentiation.

## Supplementary Material

Supplementary Figure S1 Supplementary Fig. S1 shows that knockdown of USP36 decreases the levels of mature miRNAs in HeLa and IMR-90 cells.

Supplementary Figure S2 Supplementary Fig. S2 shows that DGCR8 and Drosha can localize in the nucleolus and USP36 can colocalize with DGCR8 in the nucleolus.

Supplementary Figure S3 Supplementary Fig. S3 shows that the knockdown of Drosha does not affect USP36 interaction with DGCR8 and the knockdown of DGCR8 does not affect USP36 interaction with Drosha.

Supplementary Figure S4 Supplementary Fig. S4 shows the effects of USP36 on DGCR8 ubiquitination and that USP36 SUMOylation of DGCR8 does not depend on its DUB activity.
