# Supplementary material for: The Ubiquitin-specific Protease USP36 Associates with the Microprocessor Complex and Regulates miRNA Biogenesis by SUMOylating DGCR8
Source: Cancer Res Commun. 2023 Mar 20;3(3):459–70. doi: 10.1158/2767-9764.CRC-22-0344 (PMC10026737; doi:10.1158/2767-9764.CRC-22-0344)
Supplement: Supplementary Figure S1 — Supplementary Fig. S1 shows that knockdown of USP36 decreases the levels of mature miRNAs in HeLa and IMR-90 cells. [file crc-22-0344-s01.pdf]

Supplementary Figure S1

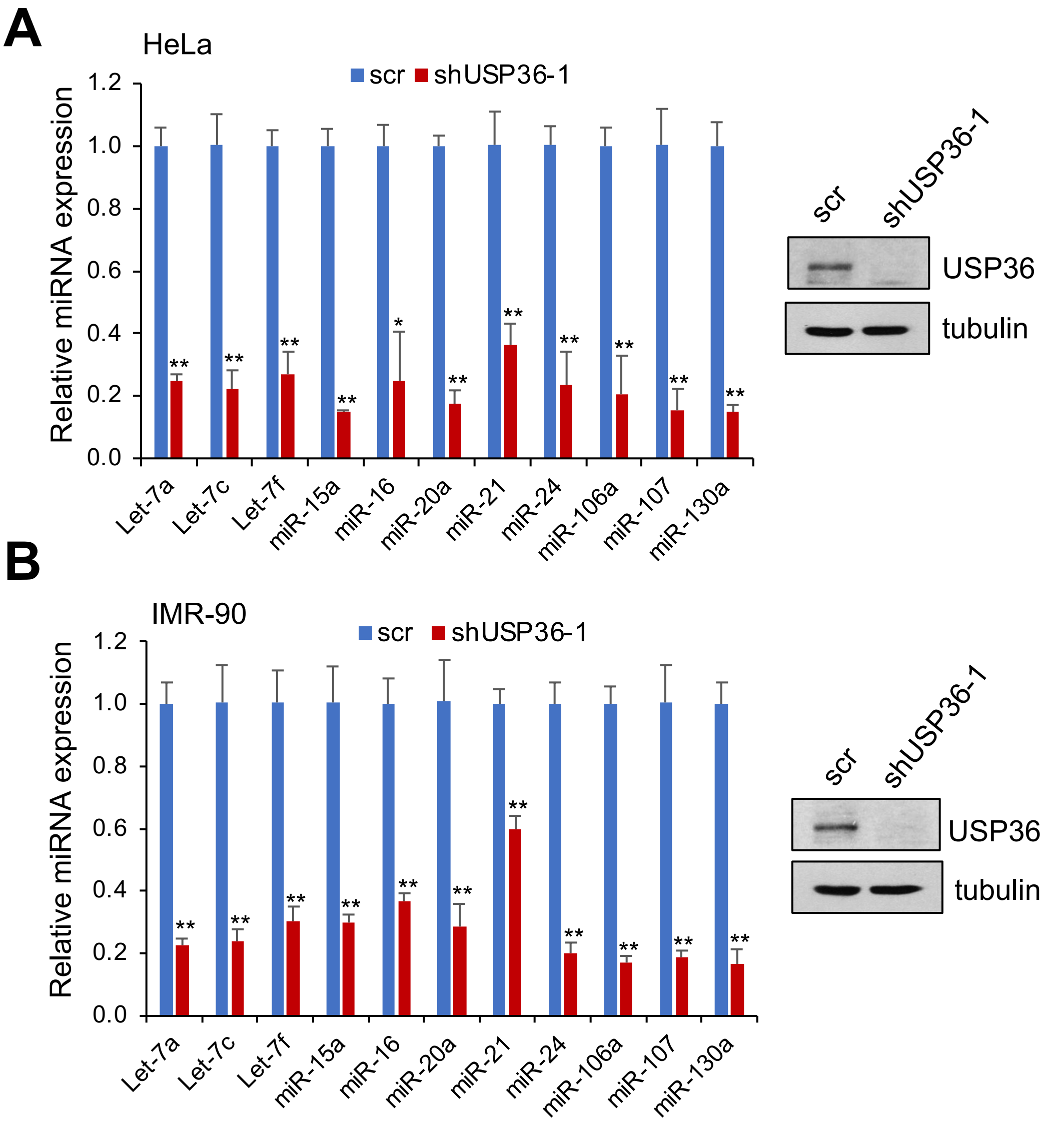

Supplementary Figure S1. Knockdown of USP36 reduces the levels of mature miRNAs in Hela and IMR-90 cells. HeLa **(A)** and IMR-90 **(B)** cells were infected with scr or USP36 shRNA-1 lentiviruses followed by RT-qPCR detection of indicated miRNAs. Shown are the relative fold changes of miRNA levels normalized to U6 RNA as an internal control in USP36 shRNA infected cells compared to scr infected cells. Data were presented as mean ± SD of three independent experiments. **, P<0.01, compared to scr control as determined by Student's t-test. The expression of USP36 assayed by IB is shown in the right.
