# Supplementary material for: The Ubiquitin-specific Protease USP36 Associates with the Microprocessor Complex and Regulates miRNA Biogenesis by SUMOylating DGCR8
Source: Cancer Res Commun. 2023 Mar 20;3(3):459–70. doi: 10.1158/2767-9764.CRC-22-0344 (PMC10026737; doi:10.1158/2767-9764.CRC-22-0344)
Supplement: Supplementary Figure S2 — Supplementary Fig. S2 shows that DGCR8 and Drosha can localize in the nucleolus and USP36 can colocalize with DGCR8 in the nucleolus. [file crc-22-0344-s02.pdf]

Supplementary Figure S2

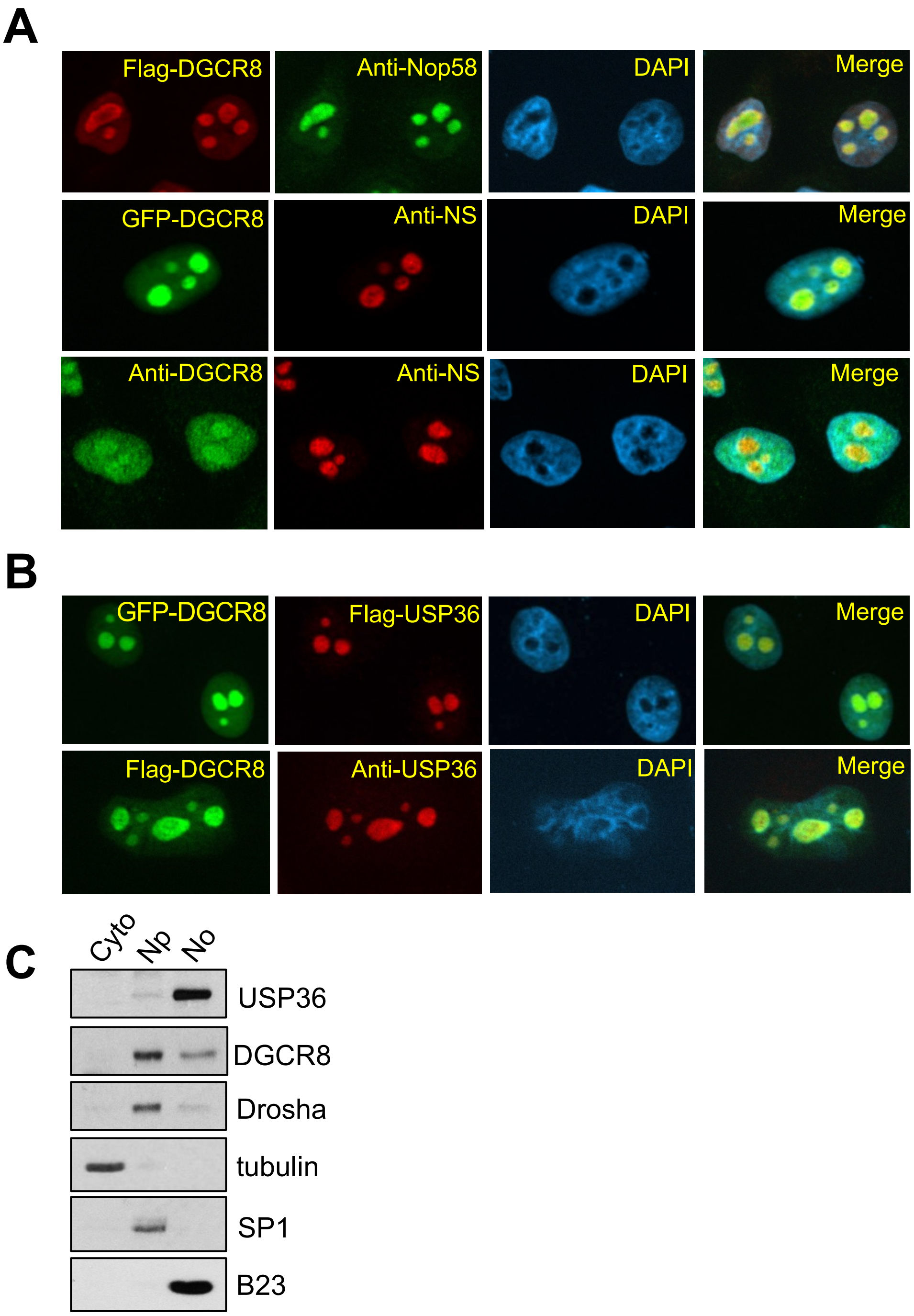

Supplementary Figure S2. Nucleolar localization of DGCR8. (A). Immunofluorescence staining of DGCR8 protein. HeLa cells were transfected with Flag-DGCR8 and stained with anti-Flag and anti-Nop58 antibodies (Top panel) or transfected with GFP-DGCR8 and stained with anti-NS antibody (middle panel) followed by DAPI staining for DNA. HeLa cells were also stained with anti-DGCR8 and anti-NS (bottom panel). **(B).** USP36 colocalizes with DGCR8 in the nucleolus. HeLa cells were transfected with GFP-DGCR8 and Flag-USP36 and stained with anti-Flag antibody (top panel) or transfected with Flag-DGCR8 and stained with anti-Flag and anti-USP36 (bottom panel). **(C).** Cell fractionation. HeLa cells were fractionated to cytoplasm (Cyto), nucleoplasm (Np) or nucleolar (No) fractions followed by IB. Tubulin, SP1 and B23 were used as cytoplasm, nucleoplasm and nucleolar markers, respectively.
