# Supplementary material for: The Ubiquitin-specific Protease USP36 Associates with the Microprocessor Complex and Regulates miRNA Biogenesis by SUMOylating DGCR8
Source: Cancer Res Commun. 2023 Mar 20;3(3):459–70. doi: 10.1158/2767-9764.CRC-22-0344 (PMC10026737; doi:10.1158/2767-9764.CRC-22-0344)
Supplement: Supplementary Figure S3 — Supplementary Fig. S3 shows that the knockdown of Drosha does not affect USP36 interaction with DGCR8 and the knockdown of DGCR8 does not affect USP36 interaction with Drosha. [file crc-22-0344-s03.pdf]

Supplementary Figure S3

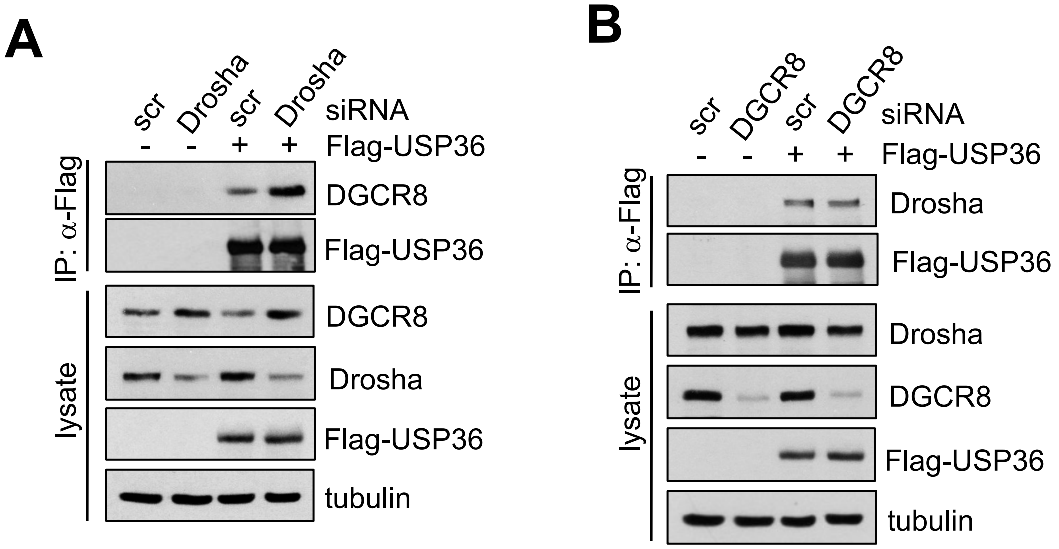

Supplementary Figure S3. USP36 interacts with DGCR8 and Drosha. (A). Knockdown of Drosha does not reduce USP36-DGCR8 binding. 293 cells transfected with Flag-USP36 together with scr control or Drosha siRNA pool were subjected to co-IP using anti-Flag antibody followed by IB. **(B)** Knockdown of DGCR8 does not reduce USP36-Drosha binding. 293 cells transfected with Flag-USP36 together with scr control or DGCR8 siRNA pool were subjected to co-IP using anti-Flag antibody followed by IB. The protein expression is shown in bottom panels.
