# Supplementary material for: The Ubiquitin-specific Protease USP36 Associates with the Microprocessor Complex and Regulates miRNA Biogenesis by SUMOylating DGCR8
Source: Cancer Res Commun. 2023 Mar 20;3(3):459–70. doi: 10.1158/2767-9764.CRC-22-0344 (PMC10026737; doi:10.1158/2767-9764.CRC-22-0344)
Supplement: Supplementary Figure S4 — Supplementary Fig. S4 shows the effects of USP36 on DGCR8 ubiquitination and that USP36 SUMOylation of DGCR8 does not depend on its DUB activity. [file crc-22-0344-s04.pdf]

Supplementary Figure S4

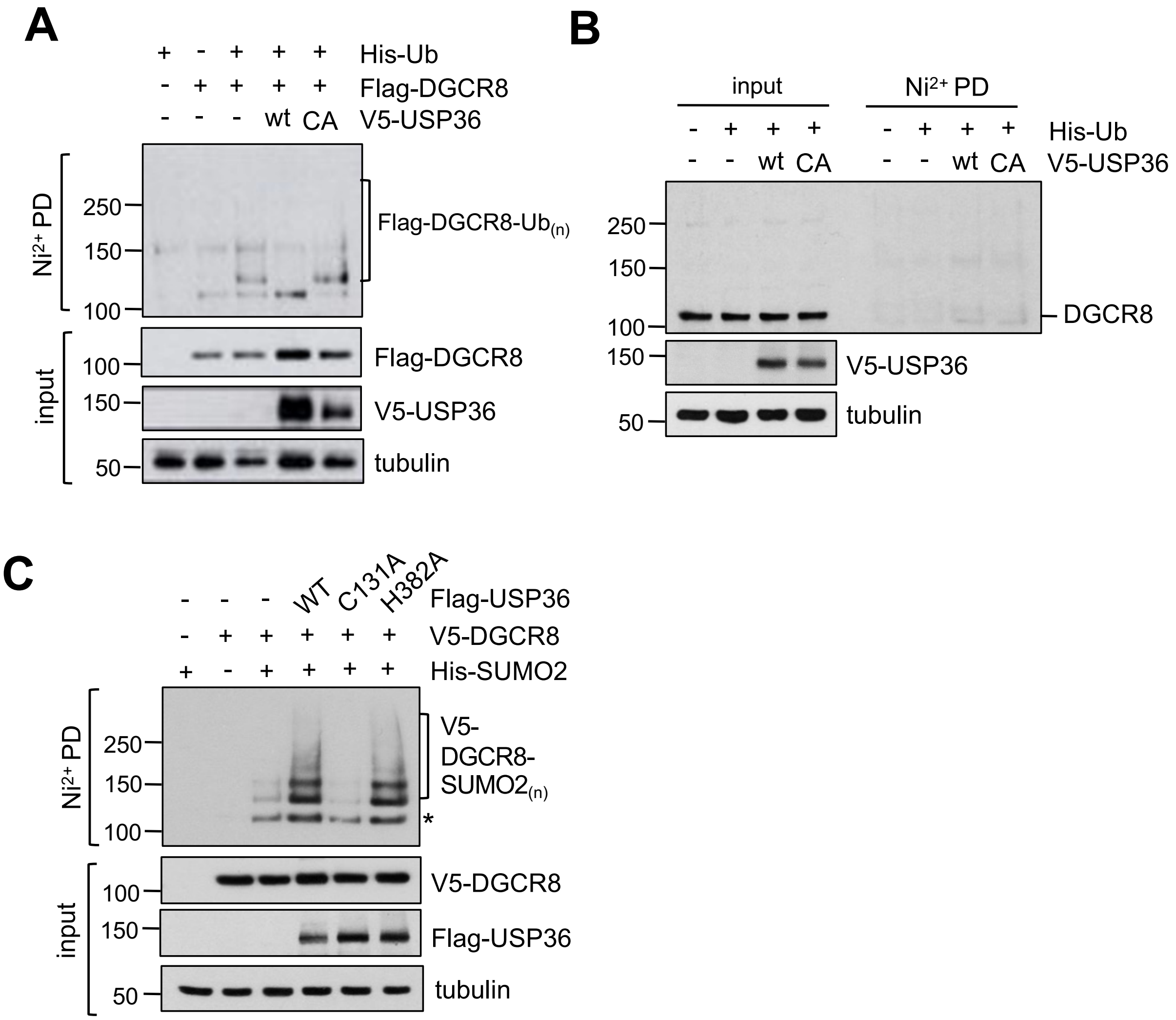

Supplementary Figure S4. **Effects of USP36 on DGCR8 ubiquitination. (A)** Overexpression of USP36 deubiquitinates exogenously expressed DGCR8. H1299 cells were transfected with His-Ub, Flag-DGCR8 together with or without wild-type (WT) V5-USP36 or its catalytically inactive C131A mutant (CA) plasmid and treated with MG132 (40 μM) for 6 hours before harvesting. The cells were then subjected to Ni^{2+}-NTA PD under denature conditions, followed by IB. The protein expression is shown in the bottom panels. **(B)**. Endogenous DGCR8 ubiquitination is under detectable. H1299 cells transfected with His-Ub with or without WT V5-USP36 or its catalytically inactive C131A mutant and treated with MG132 (40 μM) for 6 hours before harvesting. The cells were then subjected to Ni^{2+}-NTA PD under denature conditions, followed by IB. The protein expression is shown in the bottom panels. **(C)**. The effect of USP36 on promoting DGCR8 SUMOylation does not depend on its DUB activity. H1299 cells transfected with His-SUMO2, V5-DGCR8 in the absence or presence of WT USP36 or its catalytically-inactive mutants (C131A and H382A) were subjected to Ni^{2+}-NTA agarose beads pull down (PD) under denaturing conditions, followed by IB using anti-V5 antibody.
